# Editorial: Blurring boundaries: reconfiguring social and digital spaces

**DOI:** 10.3389/fsoc.2024.1420030

**Published:** 2024-05-06

**Authors:** Vincenzo Auriemma, Gennaro Iorio, Maurizio Merico, Lucas Tavares Galindo Filho

**Affiliations:** ^1^Department of Political and Social Studies, University of Salerno, Fisciano, Italy; ^2^ASCES UNITA University Center, ASCES UNITA, Cararau, Brazil

**Keywords:** blurring boundaries, social spaces, digital spaces, sociology, emotion

The COVID-19 pandemic made explicit an aspect that tacitly underlines contemporary, everyday life: the blurring boundaries between digital and social spaces and the consequent reconfiguration of identities, relationships, and institutions.

Indeed, the boundaries between the public and the private, between what happens face-to-face and on the Internet, and between the actual and the virtual are increasingly blurred. Our personal identities, social interactions, public policies, social services and, more generally, the ways in which time and space are constructed take shape in a collapsed, ambivalent context, one in which individuals are asked to define their own biographies, relationships and ties, institutions, services and policies within the blurring boundaries that constitute our digitally-mediated worlds.

Moving from these premises, this collection of articles brings together 10 articles from around the world, in order to explore the blurring of boundaries between social and digital spaces, and the consequent reconfiguration of identities, relationships and institutions. While being grounded on the sociological perspective, given the nature of the topic, the Research Topic aims at promoting a transdisciplinary approach that involves media studies, social work, education, and political science.

The papers included in the Research Topic can be assembled in four main areas of interest.

The first area refers to the context of social and digital media.

In her paper, Roberti reflects on the representation of female subjectivity in social media. The author highlights how women's images have a significant impact on the narratives and discourses of gender that populate the public sphere. In particular, Roberti focuses on the pattern of femininity represented by influencers and the connection they establish with their followers. In this way, starting from the notion of post-feminist sensibility, the author highlights how these celebrities embody its ideals in terms of self-realization, independence and empowerment, thus producing an ambivalent model of female subjectivity.

Tirocchi explores the relationship between the values that Generation Z perceives as important and the values conveyed by the media contents they consume. In this respect, on the basis of a series of focus groups conducted with 60 Italian university students the paper explores, on the one hand, the most significant values for Italian university students; on the other hand, it also investigates their favorite media and the values they believe these media platforms convey. The results reveal a substantial continuity in the values considered as important by young people, but also that young people choose or claim to appreciate authenticity and genuineness.

A second area includes papers dealing, while from different perspectives and aims, with educational issues.

The paper by Cappello and Siino discusses a case study included in the Horizon 2020 SMOOTH project. The case study adopts the emerging paradigm of educational commons with insights derived from the field of media education. The fieldwork allows to critical observe the three main dimensions of the notion of educational commons: the commoners, the commoning practices and the community and the commons goods. While presenting the preliminary findings of the case study, the paper clearly offers the chance to identify the weaknesses and the strengths of the experimental activities carried out in order to encourage young people to express “civic intentionality” and be fully “engaged citizens” in the (digital) public sphere.

Also in order to estimate the effect of the massive investment made by the Italian government in digitizing PA, in his paper Mesa analyses the impact of educational inequalities on the relationship between Italian citizens and the Public Administration (PA) during the current digital transition phase. The study is based on a web survey conducted on a sample of 3,000 citizens aged 18-64. The results confirm the influence of education on trust in PA and on the use of digital public services. More in general, the survey highlights that the educational and cultural dimension is a crucial aspect to promote digital citizenship and reveals the need to activate facilitation and accompaniment processes for citizens with less digital skills.

The third area comprises the papers that explore political and citizenship issues.

Moving from a case study of digital games, Liu explores the politics between power and digital capital embedded in the rapidly expanding Chinese cyberspace. The results allow to conclude that digital capital and power in cyberspace form a paradoxical relationship that produces four types of politics: alliance, semi-alliance, disjunction and semi-disjunction. As a result, the politics between the Chinese government and digital capitalists in cyberspace not only triggered an unexpected social transformation, but also opened a different path for Chinese digital technology.

On the other hand, Ciziceno examines the issue of climate change. The paper aims at understating which mechanisms promote individuals' environmental intentions, so as to offer a contribution to one of the main challenges that policymakers face. In this regard, the paper analyses the data from the European Values Study (wave 2017–2022) to test the hypothesis that people's life satisfaction is compatible with an environmental mindset. Results support that more satisfied people meet ecocentric values and beliefs. In other words, a growing level of social wellbeing seems to provide fertile ground for promoting ecological awareness. Therefore, according to Ciziceno, increasing social wellbeing is not only a desirable policy goal by itself, since it may also offer a fertile ground for the promotion of individuals' greener orientation and more sustainable societies.

Battista's research paper explores the interconnection between social media platforms and the contemporary political sphere, considering the multidisciplinary nature of the phenomenon. In particular, Battista insists on the shift of social media from simple sites of interaction to digital spaces that profoundly influence the political mobilization. In this respect, the paper analyses the “metamorphosis” of Vincenzo De Luca from Campania's governor to media celebrity. The article also integrates empirical data that testify to the impact of social media activities in political dynamics, offering a contribution to a deeper understanding of this complex process.

Finally, the paper by Suing et al. illustrates a study on the concept of digital citizenship from the perspectives of citizens and the media in the Andean countries. The study uses qualitative and quantitative methods, including an online survey, semi-structured interviews, and content analysis of newspaper articles. Digital citizenship refers to people's participation in managing their rights and civic engagement in the digital world. However, the actual understanding and practice of digital citizenship often differs from its theoretical definition due to perceptions, conditions, and institutional factors. Therefore, according to the authors agendas need to be revised to include the voices of the protagonists, such as Andean inhabitants who show resistance to the defense of rights or the management of social change, which could be fostered through a broader and conscious exercise of digital citizenship.

The fourth area includes two papers dealing mainly with methodological issues.

Crescenzo examines the usefulness of qualitative methods in order to understand youth experience in the digital context. The paper relies on the results of a teaching experience in which students have been asked to research online youth practices through netnography. The results show how netnography not only made it possible to analyze the contours and deep transformations of youth new digital habitats, but also offered the opportunity to observe the development of a high degree of reflexivity: indeed, students were asked to look at themselves “in the mirror,” that is to study the youth practices in which themselves are immersed from another point of view. In brief, netnography helped students to reach at once a different awareness of being young today and of being able to critically understand the contemporary digital cultural dynamics.

Catone's article explores the contemporary landscape of digitization and knowledge production, focusing on open data. Through a bibliometric analysis, the study delves into scientific trends on open data in social sciences and humanities, examining 3110 publications from 2013 to 2022. The paper reveals the interdisciplinary nature of open data studies, identifying key clusters of open data research: governance and management, data and knowledge processes, and behavioral models. The study also tracks thematic shifts over time, alongside enduring issues like data quality and transparency. More in general, the study underscores the need for interdisciplinary perspectives and critical inquiry to navigate the complexities of open data effectively. It also advocates for addressing challenges like data quality, privacy, and information overload to maximize the societal benefits of open data initiatives.

The span of subjects, questions, perspectives and methods running through the papers included in the Research Topic clearly show how rich, complex and ambitious is the challenge to explore the “blurring boundaries” through which social and digital spaces are incessantly reconfigured. For sure, they do not (and cannot) cover all the issues that are nowadays (and in perspective) on the ground. However, we believe that the main objectives of the call we launched have been fully achieved.

Specifically worth of mention are – as evident from the following papers – the constant dialogue between theory and field research, the need for mixed-methods approaches, and above all the claim for a transdisciplinary exploration of the interconnections between social and digital spaces, in particular within the context of the post-COVID-19 society.

In this respect, collecting analysis and research from various disciplinary perspectives, referring to different types of data and methods, reporting cases from different contexts and countries allowed to uncover kaleidoscopic ways of investigating, operationalizing, and evaluating the current situation, avoiding any kind of reductionism and allowing scholars to draw on the broadest possible sources.

## Author contributions

VA: Writing—original draft, Writing—review & editing. GI: Writing—original draft, Writing—review & editing. MM: Writing—original draft, Writing—review & editing. LT: Writing—original draft, Writing—review & editing.

